# Safety and effectiveness of the combination of remimazolam tosilate and propofol in gastroscopy: a multicenter, randomized controlled, single-blind clinical trial

**DOI:** 10.3389/fphar.2023.1124667

**Published:** 2023-05-31

**Authors:** Chunyan Wang, Yangzheng Gao, Jie Li, Linlin Zhang, Qing Li, Yize Li, Yuechun Lu, Jiangang Sun, Yang Zhang, Yaobei Cheng, Shidong Zhang, Guolin Wang, Yonghao Yu

**Affiliations:** ^1^ Department of Anesthesiology, Tianjin Medical University General Hospital, Tianjin, China; ^2^ Tianjin Research Institute of Anesthesiology, Tianjin, China; ^3^ Department of Anesthesiology, The Second Hospital of Tianjin Medical University, Tianjin, China; ^4^ Tianjin Fourth Central Hospital, Tianjin, China; ^5^ Tianjin Jinghai Hospital, Tianjin, China

**Keywords:** benzodiazepine (BZD), remimazolam tosilate, propofol, endoscopic sedation, gastroscopy, adverse events

## Abstract

Remimazolam tosilate (RT) is a new short-acting γ-aminobutyric acid A (GABAA) receptors agonist. However, its optimal use mode and dosage still remain unclear. This study aimed to examine the safety and effectiveness of the combination of RT and propofol in gastroscopy. This was a prospective, single-blind, randomized, multicenter, parallel-group study. All eligible 256 patients were randomized into the following 3 groups. Patients were anesthetized with propofol (Group P), RT (Group R) or the combination of RT and propofol (Group RP). The primary efficacy endpoints were: body movement score; satisfaction of gastroscopy doctors; success rate of sedation and effects on sleep status. Sedation induction time, time to be fully alert and adverse events were also recorded. The probability of complete immobility was lower in group R (33.73%) than in group P (86.67%) and RP (83.13%). The rate of doctors’ satisfaction was much lower in group R (28.92%) than in group P (77.78%) and RP (72.29%). The success rate of sedation and sleep outcome score has no difference in the three groups. The time to adequate sedation was longer in group RP (77.27 ± 18.63 s) than in group P (64.47 ± 24.36 s), but much shorter than that in group R (102.84 ± 46.43s). The time to be fully alert was shorter in group R (6.30 ± 1.52 min) and RP (6.54 ± 1.13 min) than in group P (7.87 ± 1.08 min). The proportion of sedative hypotension was significantly higher in group P (41.11%) than in group R (1.20%) and group RP (3.61%) (*p* < 0.001). The incidence of respiratory depression was much higher in group P (17.78%) than in group R (no patient) and group RP (1.2%). The incidence of adverse events was lower in groups R (4.82%) and RP (9.64%) than in group P (31.11%). The combination of RT and propofol takes effect quickly, makes patients alert quickly, provides a sufficient depth of sedation, reduces body movement, does not inhibit circulation and respiratory function, does not affect sleep, and is the preferred mode for gastroscopy doctors and anesthesiologists.

## 1 Introduction

Gastroscopy, enteroscopy, and other endoscopic examinations are often difficult for patients to cooperate with and leave an unpleasant impression on patients (Goudra et al., 2020). The use of sedatives can improve this situation, make these examinations proceed more smoothly, and relieve patients’ pain (Moon et al., 2017). At present, midazolam and propofol are commonly used as anesthetic and sedative drugs for outpatient surgery such as gastrointestinal endoscopy (Triantafillidis et al., 2013). These two anesthetic and sedative drugs are mostly used for general anesthesia (Morimoto, 2022). Although they can basically meet the sedation needs of outpatient surgery, some problems still cannot be ignored. For example, midazolam has a long duration of action and slow recovery from anesthesia, and is mainly metabolized by cytochrome P450 3A4 enzyme in the liver. The metabolites of midazolam still have a sedative effect, making the sedation time of midazolam uncontrollable (Ruesch et al., 2012). Propofol offers deep sedation with quick onset and recovery, but it may lead to cardiovascular and respiratory system depression and hypoxemia, including the possibility of causing cardiopulmonary failure and emergency intubation. Continuous monitoring of vital signs and respiration is required when propofol is used (Rex et al., 2009; Wernli et al., 2016).

Remimazolam tosilate (RT) is a new ultrashort-acting benzodiazepine having a high affinity for the GABAA receptor (Pambianco et al., 2016; Wesolowski et al., 2016). The GABAA receptor is a ligand-gated chloride channel. When RT acts on the GABAA receptor, it can increase the chloride permeability of the nerve cell membrane and chloride influx, causing the hyperpolarization of the nerve cell membrane, thus inhibiting neuronal activity and playing a sedative role (Rogers and McDowell, 2010). Some studies (Antonik et al., 2012; Zhou et al., 2015) proved the varied advantages of RT, such as rapid-onset, controllable inhibition of cardiovascular and respiratory systems, inactive metabolites through plasma esterase metabolism, low potential of drug interaction, and reversibility. Therefore, it can be used for sedation outside the operating room, such as bronchoscopy (Pastis et al., 2019), hysteroscopy (Zhang et al., 2021), molar extraction (Zhao et al., 2022), and other endoscopic examinations (Rex et al., 2018).

However, the dosage of RT varies greatly for gastroscopy. A single dose of 0.10–0.20 mg/kg was given in some clinical trials (Borkett et al., 2015; Tan et al., 2022). In another clinical trial, 5 mg RT was given as the initial dose (Chen et al., 2021). Therefore, its optimal use mode and dosage are still unclear. A previous multicenter project (Chen et al., 2021) confirmed that RT combined with fentanyl helped safely accomplish gastroscopy. The success rate of sedation was 97.34%. However, the incidence of patient’s body movements was high during the examination, and gastroscopy doctors were not satisfied with the anesthetic effect. Whether the combination of RT and propofol could maximize their advantages was not reported. Therefore, this study mainly examined the safety and effectiveness of the combination of RT and propofol in gastroscopy, thus providing a theoretical basis for the clinical use of RT in the future.

## 2 Materials and methods

### 2.1 Ethics and registration

This study was approved by the Clinical Research Ethics Committee of the Tianjin Medical University General Hospital (IRB2020-YX-040-01) and registered at http://www.chictr.org.cn (28 September 2020; ChiCTR2000038694). The study protocol followed the consolidated standards of reporting trial (CONSORT) guidelines. The whole trial was conducted according to the Declaration of Helsinki and the International Conference on Harmonization of Good Clinical Practice. All participating centers obtained approval from the institutional review board for participation. Written informed consent was obtained from patients undergoing upper gastrointestinal endoscopy before the start of any protocol-specified procedures.

### 2.2 Overall design

This was a prospective, single-blind, randomized, multicenter, parallel-group study assessing the efficacy and safety of RT (Jiangsu Hengrui Pharmaceutical Co., Ltd., China), propofol (AstraZeneca Pharmaceuticals Co., Ltd., United States), and the combination of RT and propofol. The trial was performed at four centers in China.

### 2.3 Participants

The inclusion criteria were as follows (Goudra et al., 2020): 18≤ age ≤70 years, no sex limitation (Moon et al., 2017); patients undergoing routine gastroscopy (Triantafillidis et al., 2013); American Society of Anesthesiologists.

[ASA] classification I or II (Morimoto, 2022); 18 kg/m^2^ < body mass index (BMI) < 30 kg/m^2^ (Ruesch et al., 2012); surgery duration no more than 30 min (Rex et al., 2009); no sleep disorder; and (Wernli et al., 2016) clear understanding and voluntary participation in the study and signed informed consent form. The exclusion criteria were as follows (Goudra et al., 2020): need for endoscopic diagnosis and treatment techniques with complicated surgery (pancreaticocholangiography, endoscopic ultrasonography, endoscopic mucosal resection, endoscopic submucosal dissection, intraoral endoscopic myotomy, and so on) (Moon et al., 2017); need for intubation (Triantafillidis et al., 2013); patients judged as having difficulty in managing the respiratory tract (modified Mallampati score grade IV) (Morimoto, 2022); history of anemia or thrombocytopenia (Ruesch et al., 2012); history of abnormal liver function (Rex et al., 2009); history of abnormal renal function (Wernli et al., 2016); history of drug abuse and/or alcoholism within 2 years before screening [Alcoholism meant that the average daily alcohol consumption exceeded 2 units (1 unit = 360 mL of beer or 45 mL of Baijiu or 150 mL of wine with 40% alcohol)] (Pambianco et al., 2016); patients with hypertension whose blood pressure had not been satisfactorily controlled using antihypertensive drugs (sitting systolic blood pressure ≥160 mm Hg and/or diastolic blood pressure ≥100 mm Hg while screening) (Wesolowski et al., 2016); sitting systolic blood pressure ≤90 mm Hg during screening (Rogers and McDowell, 2010); pregnant or lactating women (Antonik et al., 2012); allergy or contraindication to benzodiazepines, opioids, propofol, lidocaine, and other drugs and their components (Zhou et al., 2015); participation in the drug clinical trial as a subject in the last 3 months; and (Pastis et al., 2019) investigator considering the participation of patients in the trial inappropriate.

### 2.4 Randomization

All eligible patients were randomized into one of the three groups in the ratio of 1:1:1. They were anesthetized with propofol (group P), RT (group R), or RT combined with propofol (group RP) within 24 h prior to upper gastrointestinal endoscopy. The central randomization method was used for grouping, with each center competing for admission. The centralized randomization software “91trail” of Aisha medicine was used for the randomization program. The evaluation investigator and the administration investigator were set up in this trial. Besides blinding the patients, the evaluation investigator was also blinded during the whole trial process.

### 2.5 Study procedures and drug administration

The complete routine preparation before gastroscopy included fasting for at least 6 h and discontinuation of water intake for at least 2 h before the surgery. Before the sedation induction, the patients took 10 g lidocaine hydrochloride glue in the throat for about 5 min and slowly swallowed it till they felt that the tongue was enlarged and the throat was numb. Then, the administration investigator administered butorphanol tartrate (Jiangsu Hengrui Pharmaceutical Co., Ltd., China) 5 μg/kg intravenously. After 3 min of butorphanol tartrate administration, an initial intravenous dose of propofol 1.5 mg/kg for group P, RT 7.5 mg for group R, and RT 3.75 mg and propofol 0.75 mg/kg for group RP was administered to patients for the induction of sedation. The upper gastrointestinal endoscopy was initiated when adequate sedation [Modified Observer’s Assessment of Alertness/Sedation (MOAA/S) score ≤3] (Borkett et al., 2015) was achieved. If patients did not achieve adequate sedation after the initial dose of propofol or RT, they were given a maximum of five doses of propofol (0.5 mg/kg each time) in group P, RT (3.75 mg each time) in group R, and propofol (0.25 mg/kg each time) and RT (1.25 mg each time) in group RP. For the maintenance phase of sedation, adequate sedation (MOAA/S score ≤4) was maintained using propofol (0.5 mg/kg each time) in group P, RT (3.75 mg each time) in group R, and propofol (0.25 mg/kg each time) and RT (1.25 mg each time) in group RP; and the time interval was required to be more than 1 min. From the end of the initial dose of the test drug, if the number of additional administrations was more than five times during a 15-min period, it was considered as sedation failure, and then the investigator could decide on sedation remedial measures. Further, the patient was given oxygen inhalation (2–4 L/min) before the start of butorphanol tartrate administration until the patient completely woke up after the surgery.

### 2.6 Clinical outcomes

The primary efficacy endpoints included the following (Goudra et al., 2020): Body movement, including retching, swallowing, and limb movement): I, completely immobile; II, slight body movement; III, general body movement (not affecting inspection); and IV, serious body movement (affecting the inspection and forcing the inspection to be interrupted) (Moon et al., 2017). Satisfaction of gastroscopy doctors with anesthesia effect: I, very satisfied; II, satisfied; III, generally dissatisfied; IV, majorly dissatisfied; and V, very dissatisfied (Triantafillidis et al., 2013). Success rate of sedation: Sedation success was defined as follows (Goudra et al., 2020): the whole operation process under gastroscopy completed (Moon et al., 2017); no sedative remedy given; and (Triantafillidis et al., 2013) no more than five additional doses administered within a 15-min period after the initial dose of the test drug was administered. The success rate of sedation was defined as the proportion of participants who succeeded in sedation in this group (Morimoto, 2022). Effects on sleep status, assessed using the Pittsburgh Sleep Quality Index (PSQI) (Buysse et al., 1989): The PSQI assessed subjective sleep quality and sleep disturbances over the previous month. It consisted of 19 items evaluated over 7 domains including subjective sleep quality, sleep latency, sleep duration, habitual sleep efficiency, sleep disturbances, use of sleep medications, and daytime dysfunction. The domains were scored on a 0–3 scale where 3 indicated severe impairment. The seven subscale scores were then totaled to provide a global PSQI score, which had a range of 0–21, with higher scores indicating worse sleep quality.

The secondary outcomes included the following (Goudra et al., 2020): Sedation induction time: It was defined as the time from the initial dose of RT or propofol to the first time the MOAA/S score was ≤3 (Moon et al., 2017). Time to be fully alert: the time from stopping the use of RT or propofol to the time when the patient was fully awake (the MOAA/S score for three consecutive times was 5) (Triantafillidis et al., 2013). Sedative hypotension: the decrease in systolic blood pressure during the period from the beginning of the administration of RT or propofol to the patient’s full consciousness exceeding 20% before sedation or the systolic blood pressure dropping to ≤80 mm Hg. Sedative hypotension to be treated: the hypotension during the period from the beginning of the administration of remimazolam or propofol to the patient’s full consciousness needing treatment with vasopressor drugs. The timing and types of the vasopressors were decided by anesthesiologist (Morimoto, 2022). The incidence of respiratory depression during sedation: It was defined as the incidence of respiratory rate <8 times/min and/or blood oxygen saturation <90% during the period from the initial dose of RT or propofol to the patient’s full consciousness.

### 2.7 Other observations

(1) MOAA/S score: MOAA/S score was recorded 1(T1), 1.5 (T1.5), 2(T1.25), 2.5(T2.5), and 3 (T3) min after the initial dose of RT or propofol was administered (recorded as 0 min (T0) at this time), and then every 1 min until the MOAA/S score reached 5 points for three consecutive times. These time points were recorded as T4, T5, T6,T7, T8 and so on.
(2) Adverse events: nausea, vomiting, headache, dizziness, somnolence, chills, and injection pain.

### 2.8 Sample size

Our preliminary study showed that the probability of absolute immobility of propofol during gastroscopy was about 86% in our department. We assumed that the incidence rate of absolute immobility in groups RP and P was about 86%, using a noninferiority test with *α* = 0.05 and *β* = 20%, The non-inferiority margin(δ) was 15%. Under these assumptions, each group had a minimum of 67 patients. Considering the potential loss of follow-up (15%), we increased the sample size of each group to 78.

### 2.9 Statistical analysis

Calculations were carried out using Statistical Package of Social Science, Windows version 25 (Statistical Package for the Social Sciences (SPSS), IBM). Descriptive statistics were used to evaluate all data, continuous data were presented with means and standard deviations while numerical data with median and interquartile ranges. Continuous data were compared using ANOVA, if significantly different then using LSD multiple comparison analysis method. Numerical data were compared using a Chi-square test, or the Fisher exact test, as appropriate. All statistical tests were two-sided, and *p* value of < 0.05 was considered statistically significant.

## 3 Results

### 3.1 Patient characteristics

**FIGURE 1:**
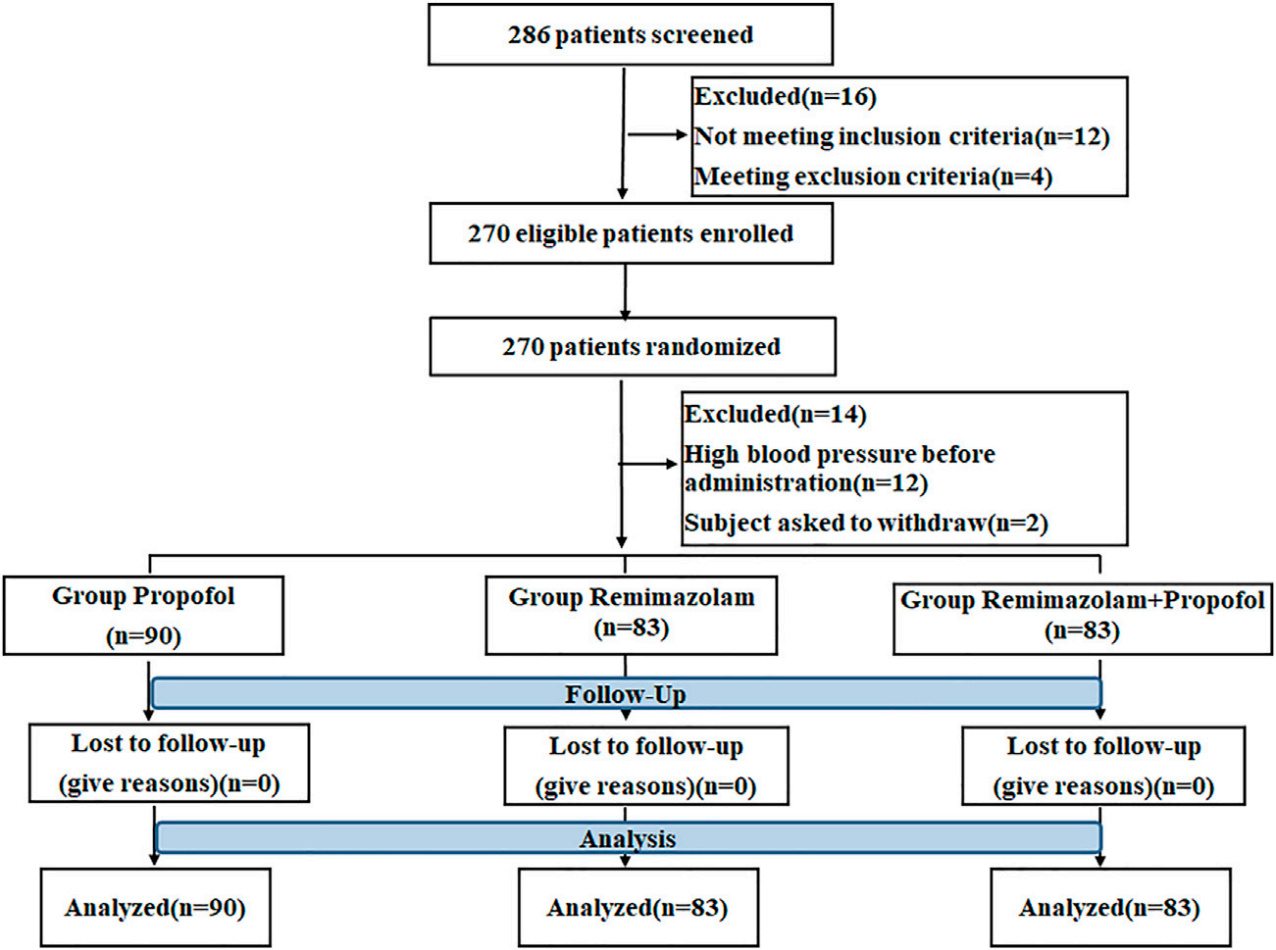
Schematic illustration of the study flow.

A total of 286 patients from 4 centers who underwent gastroscopy were selected for the study. Of these, 16 patients were excluded because they did not meet the inclusion criteria or meet exclusion criteria. 270 patients were randomized in the ratio of 1:1:1, 12 patients were excluded because of high blood pressure before administration and 2 patients asked to withdraw. So a total of 256 patients were randomly divided into propofol (n = 90), RT (n = 83), and the combination of RT and propofol groups (n = 83). No patients were dropped out during drug administration and the follow-up period. The detailed study flow chart is shown in Figure 1.

### 3.2 Baseline characteristics

The three groups had a good balance in age, sex, ASA score, BMI, and relevant medical history (allergy, alcohol consumption, hypertension, and diabetes) (Table 1). No significant difference in vital signs was observed at baseline among the three groups, including body temperature, systolic blood pressure, diastolic blood pressure, respiratory rate, heart rate, and blood oxygen saturation (all *p* > 0.05).

**TABLE 1:** Baseline characteristics.

Characteristic	P (n = 90)	R (n = 83)	RP (n = 83)	*p*
Age[y,(X ± S)]	49.19 ± 11.39	46.37 ± 12.48	48.63 ± 12.56	0.274
Gender(Male: Female)	41:49	40:43	39:44	0.941
ASA class(Ⅰ: Ⅱ)	28:62	28:55	26:57	0.921
Height [cm,(X ± S)]	167.13 ± 6.40	166.36 ± 8.08	167.40 ± 8.39	0.662
Weight[kg,(X ± S)]	66.56 ± 12.84	67.92 ± 9.35	66.62 ± 11.03	0.673
BMI[kg/m^2^,(X ± S)]	23.72 ± 3.74	24.48 ± 2.36	23.68 ± 2.85	0.167
History, yes (%)				
allergy	4(4.44)	6(7.23)	5(6.02)	0.736
Alcohol	21(23.33)	19(22.89)	18(21.69)	0.965
Hypertension, diabetes	11(12.22)	7(8.43)	10(12.05)	0.673
Temperature[°C,(X ± S)]	36.40 ± 0.17	36.40 ± 0.17	36.37 ± 0.15	0.437
Blood pressure[mmHg,(X ± S)]				
Systolic pressure	122.28 ± 13.05	124.16 ± 10.76	121.51 ± 11.91	0.343
Diastolic pressure	76.57 ± 7.34	74.37 ± 7.08	75.4 ± 7.70	0.150
Respiratory rate [times/min,(X ± S)]	16.48 ± 6.03	15.94 ± 1.14	16.06 ± 1.41	0.605
Heart rate[times/min,(X ± S)]	73.66 ± 10.01	73.75 ± 6.74	74.66 ± 6.01	0.654
SpO_2_[%,(X ± S)]	98.48 ± 0.62	98.57 ± 0.63	98.48 ± 0.67	0.599

### 3.3 Clinical outcomes

#### 3.3.1 Primary efficacy endpoints

##### 3.3.1.1 Body movement score

The probability of complete immobility was 86.67% (group P), 33.73% (group R), and 83.13% (group RP). The probability of complete immobility in group R was lower than that in group P (p < 0.001) and RP (*p* < 0.001), with no statistically significant difference between groups P and RP (*p* > 0.05). The median of the three groups showed complete immobility (group P), slight body movement (group R), and complete immobility (group RP) (Table 2).

**TABLE 2:** Comparison of body movement scores among three groups.

N = 256	P group(n = 90)	R group(n = 83)	RPgroup(n = 83)	*p*
I (n[%])	78(86.67)	29(33.73)	69(83.13)	<0.001
II (n[%])	12(13.33)	42(50.60)	14(16.87)	
III(n[%])	0	12(14.46)	0	
IV(n[%])	0	0	0	

I, completely immobile; II, slight body movement; III, general body movement (not affecting inspection); IV, serious body movement (affecting the inspection and forcing the inspection to be interrupted).

B, the violin plot of body movement scores.

**FIGURE 2:**
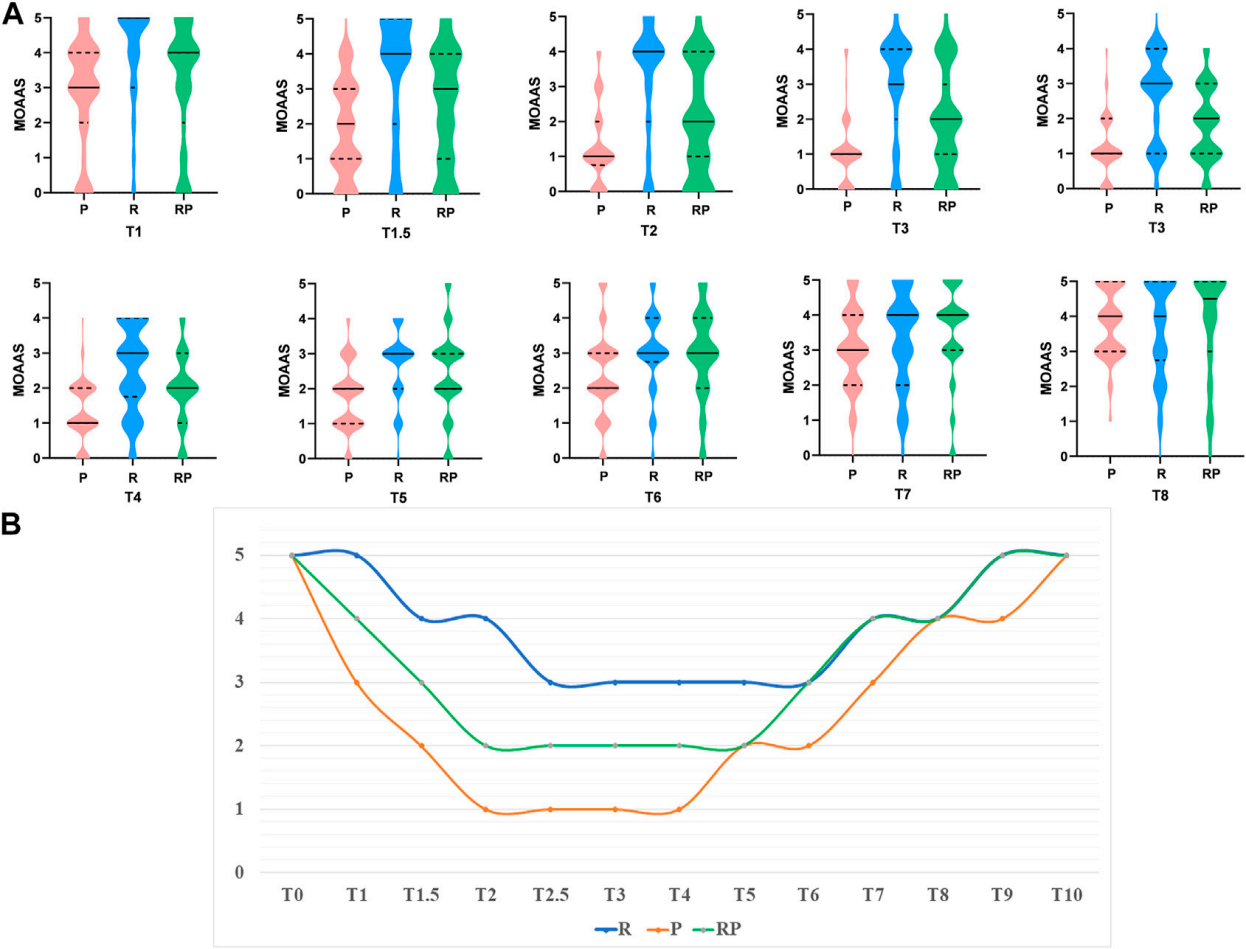
The results of MOAA/S score. **(A)**. The Violin Plot of MOAA/S score among three groups at different time point (T1–T8). **(B)**. The medium of MOAA/S score among three groups at different time point.

Moreover, we recorded the MOAA/S score at each time point (T1–T8) (Figure 2A). Figure 2B shows that the depth of sedation could achieve MOAA/S = 2 in group RP during the maintenance period (MOAA/S = 1 in group P and MOAA/S = 3 in group R).

##### 3.3.1.2 Satisfaction of gastroscopy doctors with anesthesia effect

The rate at which the gastroscopy doctors were very satisfied with the anesthetic effect was 77.78% (group P), 28.92% (group R), and 72.29% (group RP) (Table 3). The rate of gastroscopy doctors achieving very satisfaction in group R was much lower than that in groups P (p < 0.001) and RP (*p* < 0.001). The median of the three groups showed very satisfied (group P), satisfied (group R), and very satisfied (group RP) (Table 3).

**TABLE 3:** Comparison of physician satisfaction among three groups.

N = 256	P group(n = 90)	R group(n = 83)	RP group(n = 83)	*χ* ^*2*^	*P*
I(n[%])	70(77.78)	24(28.92)	60(72.29)		
II(n[%])	20(22.22)	50(60.24)	23(27.71)		
III(n[%])	0	7(8.43)	0	58.592	<0.001
IV(n[%])	0	1(1.20)	0		
V(n[%])	0	1(1.20)	0		

I, very satisfied; II, Satisfied; III, Generally dissatisfied; IV, Major dissatisfaction; V, very dissatisfied.

##### 3.3.1.3 Success rate of sedation

The success rate of sedation in the three groups was 100% (group P), 97.59% (group R), and 100% (group RP) (Table 4).

**TABLE 4:** Comparison of sedation success rate among three groups.

N = 256	P Group (n = 90)	R Group (n = 83)	RP group (n = 83)	*p*
Sedation success (n[%])	90(100.00)	81(97.59)	83(100.00)	0.122

##### 3.3.1.4 Effects on sleep status

The sleep status was assessed using PSQI. The sleep outcome score did not change at baseline and 7 (D7) and 30 (D30) days after gastroscopy (Table 5).

**TABLE 5:** Pittsburgh Sleep Quality Index(PSQI)in the three groups.

N = 256	P group(n = 90)	R group(n = 83)	RPgroup(n = 83)	*p*
D0(n(%)				
PSQI≤5	54(60)	49(59.04)	55(66.27)	
PSQI[6,10]	36(40)	32(38.55)	26(31.32)	
PSQI[11,15]	0	2(2.41)	2(2.41)	0.237
D7[n(%)]				
PSQI≤5	58(64.44)	48(57.83)	56(67.47)	
PSQI[6,10]	32(35.56)	35(42.17)	25(30.12)	
PSQI[11,15]	0	0	2(2.41)	0.165
D30[n(%)]				
PSQI≤5	61(67.78)	52(62.65)	57(68.67)	
PSQI[6,10]	29(32.22)	31(37.35)	24(28.92)	
PSQI[11,15]	0	0	2(2.41)	0.253
*p*	0.551	0.817	0.998	

#### 3.3.2 Secondary outcomes

##### 3.3.2.1 Sedation induction time

The time to adequate sedation in the three groups was 64.47 ± 24.36 s (group P), 102.84 ± 46.43 s (group R), and 77.27 ± 18.63 s (group RP) (Table 6). The time to adequate sedation in group R was longer than that in group RP (*p* < 0.001). The time to adequate sedation in group RP was longer than that in group P (*p* = 0.009). (Table 6).

**TABLE 6:** Comparison of sedation induction time and recovery time among three groups.

N = 256	P group(n = 90)	R group(n = 83)	RP group(n = 83)	*p*
Time to sedation[sec,(X ± S)]	64.47 ± 24.36	102.84 ± 46.43	77.27 ± 18.63	<0.001
Time to fully alert[min,(X ± S)]	7.87 ± 1.08	6.30 ± 1.52	6.54 ± 1.13	<0.001

##### 3.3.2.2 Time to be fully alert

The time to be fully alert in the three groups was 7.87 ± 1.08 min (group P), 6.30 ± 1.52 min (group R), and 6.54 ± 1.13 min (group RP) (Table 6). The time to be fully alert in groups R (*p* < 0.001)and RP (*p* < 0.001)was shorter than that in group P. However, the time to be fully alert in groups R and RP showed no significant difference (*p* = 0.217, Table 6).

##### 3.3.2.3 Sedative hypotension

The incidence of hypotension (Table 7) was significantly higher in group P (41.11%) than in groups R (4.82%, *p* < 0.001) and RP (10.84%, *p* < 0.001). No significant difference was observed in the incidence of hypotension between groups R and RP (*p* = 0.149). The incidence of treatment-related hypotension in group P was 8.89%, which was much higher than that in groups R (no patient) and RP (1.2%, *p* < 0.023). However, no significant difference was found in hypotension requiring treatment between groups R and RP (*p* = 0.316).

**TABLE 7:** Comparison of the incidence of hypotension, the incidence of hypotension requiring treatment and the incidence of respiratory depression among the three groups.

N = 256	P group(*n* = 90)	R group(*n* = 83)	RP group(*n* = 83)	*p*
Incidence of hypotension (n[%])	37(41.11)	4(4.82)	9(10.84)	<0.001
Incidence of hypotension requiring treatment (n[%])	8(8.89)	0	1(1.20)	0.002
Incidence of respiratory depression (n[%])	16(17.78)	0	1(1.20)	<0.001

##### 3.3.2.4 Incidence of respiratory depression during sedation

Besides, the incidence of respiratory depression in group P was 17.78%, which was much higher than that in groups R (no patient) and RP (1.2%, *p* < 0.001).

### 3.4 Adverse events

Table 8 summarizes the incidence of adverse events. In the propofol group, 28 patients (31.11%) had adverse events of different types, including nausea (5 patients), vomiting (2 patients), headache (2 patients), dizziness (2 patients), somnolence (1 patient), and injection pain (23 patients). In group R, 4 patients had adverse reactions: nausea (one patient), dizziness (two patients), and injection pain (one patient). In group RP, 8 patients had adverse reactions, including nausea (three patients), dizziness (two patients), somnolence (one patient), and injection pain (one patient).

**TABLE 8:** Comparison of the adverse events among the three groups.

*n* = 256	P (*n* = 90)	R (*n* = 83)	RP(*n* = 83)	
nausea (n[%])	5(5.56)	1(1.20)	3(3.61)	
vomit (n[%])	2(2.22)			
headache (n[%])	2(2.22)			
dizziness (n[%])	2(2.22)	2(2.41)	2(2.41)	
somnolence (n[%])	1(1.11)		1(1.20)	
chills (n[%])				
injection pain (n[%])	23(25.56)	1(1.20)	6(7.23)	
Total patient(n[%])	28(31.11)	4(4.82)	8(9.64)	
*p*				0.000

## 4 Discussion

This study was designed to evaluate the efficacy and safety of propofol (group P), RT (group R), and the combination of RT and propofol (group RP) in patients undergoing gastroscopy. The findings were as follows (Goudra et al., 2020). The body movement was effectively inhibited in groups P and RP (Moon et al., 2017). The gastroscopy doctors had a higher level of satisfaction in groups P and RP (Triantafillidis et al., 2013). No statistically significant difference in the success rate of sedation was found among the three groups (Morimoto, 2022). The sleep quality was unaffected in the three groups (Ruesch et al., 2012). The induction time was short in groups P and RP (Rex et al., 2009). The recovery time was short in groups R and RP (Wernli et al., 2016). The incidence of hypotension and respiratory depression was lower in groups R and RP than in group P.

Previous studies confirmed that RT could be safely and effectively used for sedation in outpatient surgeries, such as gastrointestinal endoscopy (Rex et al., 2018; Lu et al., 2022; Xin et al., 2022), bronchoscopy (Pastis et al., 2019), tooth extraction (Zhao et al., 2022), hysteroscopy (Zhang et al., 2021), or induction and maintenance of general anesthesia (Zhou et al., 2020; Mao et al., 2022). RT could also provide enough sedative effect and safety for special patients, such as elderly patients (Liu et al., 2022; Tan et al., 2022) and patients with liver cirrhosis (Cao et al., 2022). However, we found that 5 mg of RT (up to 12.5 mg) could help complete the gastroscopic process, but the patients had more body movements, leading to potential risks of tissue damage. The gastroscopy doctors were not satisfied. Propofol took effect quickly and led to less body movement, but it induced circulatory and respiratory depression (Rex et al., 2009; Wernli et al., 2016). Therefore, allowing the two drugs to exert their respective effects, that is, rapid onset and rapid awakening, while not affecting circulation and respiration, was a key issue to be explored in this clinical trial.

This trial was divided into three groups: group P: the initial administration dose of propofol was 1.5 mg/kg; group R: the initial administration dose of RT was 7.5 mg because our previous study (Chen et al., 2021) (5 mg RT used for gastroscopy) showed that the median number of additional sedative medications was 1; group RP: RT 3.75 mg and propofol 0.75 mg/kg were used on the basis of the results of the pretest.

The results of this study showed that the probability of being completely immobile during gastroscopy was not significantly different between group P and RP. However, the probability of being completely immobile in group R was significantly lower than that in group P (*p* < 0.001) and RP (*p* < 0.001). The median values of the body movement indices in the three groups showed complete immobility (group P), slight body movement (group R), and complete immobility (group RP). Figures 2A, B shows that the depth of sedation could achieve MOAA/S = 2 in group RP compared with groups P (MOAA/S = 1) and R (MOAA/S = 3) during the maintenance period, which proved that the combination of RT and propofol could provide deeper depth of anesthesia and effectively inhibit the body movement of patients and avoid unnecessary tissue damage.

At the same time, the extremely satisfactory rate of the gastroscopy doctors regarding sedation was 77.78% (group P) and 72.29% (group RP), which was significantly higher than that in group R (28.92%, *p* < 0.001). The rates of successful sedation in groups P (100%) and R (97.59%) were consistent with the results of previous phase III trials. Also, the rate of successful sedation was 100% in group RP, demonstrating that the gastroscopy could be successfully completed in the three groups. Despite no statistically significant difference in the sedation success rates among the three groups, the gastroscopy doctors preferred to choose the sedation scheme with less body movement (group P and RP).

We also used PSQI to evaluate the sleep status of patients. No significant change was observed in the three groups of patients before gastroscopy and 7 and 30 days after gastroscopy, indicating that propofol or RT did not affect the short-term and long-term sleep quality of patients.

A large number of people need endoscopic examinations in China. They require sedatives to take effect and wake up quickly. Therefore, we also recorded the sedation and awakening times of patients in the three groups. The sedation time in group RP (77.27 ± 18.63 s) was longer than that in group P (64.47 ± 24.36 s), but much shorter than that in group R (102.84 ± 46.43 s) and also shorter than that of the previous commonly used sedatives, such as dexmedetomidine (Mason et al., 2011) (8.6–13 min) or midazolam (Triantafillidis et al., 2013) (16 min). As the metabolism of RT is too fast, the dosage needs to be adjusted during gastroscopy. This study confirmed that 65.1% of patients in group R needed one supplement dose, and 24.1% of patients needed two or more supplement doses to achieve a sufficient sedation effect for endoscopy. The initial low dose of RT (7.5 mg) might not be sufficient to induce faster sedation. Therefore, the optimization of the initial loading dose still needs further exploration. However, 50.6% of patients did not need an additional dose, and 44.6% of patients needed one additional dose in the group RP. This showed that RP combination had the advantage of rapid onset for short procedures, such as gastrointestinal endoscopy.

The time to being fully alert in group R (6.30 ± 1.52 min) was significantly lower than that in group P (7.87 ± 1.08 min) in this study. The time to being fully alert in group RP was 6.54 ± 1.13 min, which was significantly lower than that in group P and showed no difference compared with group R. It proved that propofol led to deep sedation and the awakening time was longer. The combination of RT and propofol could reduce propofol dosage and, at the same time, achieve sufficient sedation. Therefore, the awakening time was significantly shortened.

We also recorded the changes in blood pressure, heart rate, respiratory rate, and SpO_2_ after RT or propofol injection to evaluate the safety of the three sedation methods. The results showed that the incidence of hypotension and hypotension requiring treatment in group P was higher than that in group R, and the incidence of respiratory depression was higher than that in group R. The incidence of hypotension and respiratory depression in group RP was lower than that in group P. It indicated that the administration mode in group RP was safe. Due to the definition of “Sedative hypotension to be treated” is still controversial. So in this trial, “Sedative hypotension to be treated” is decided by anesthesiologist. Because the criteria for administering vasopressors by the same anaesthesiologist was consistent, there was no bias among groups.

At the same time, we also recorded the adverse events. In this study, 28 patients (31.11%) in group P had adverse events of different types, including nausea, vomiting, headache, dizziness, somnolence, and injection pain (23 patients). However, the combination of RT and propofol could significantly decrease the incidence of adverse events, especially injection pain (one patient), increasing satisfaction level of patients.

Despite important findings, this study also had some limitations. For example, we only selected the drug dosage based on the previous results in group RP and carried out preliminary experiments to verify the effectiveness. However, further studies are needed to explore a better drug matching technique. This study did not examine patients aged more than 70 years, patients aged less than 18 years, or patients with a BMI greater than 30, which would be the focus of our subsequent studies.

## 5 Conclusion

The combination of RT and propofol took effect quickly, made patients alert quickly, provided a sufficient depth of sedation, reduced body movement, did not inhibit circulation and respiratory function, did not affect sleep quality, and was the preferred mode for gastroscopy doctors and anesthesiologists.

## Data Availability

The raw data supporting the conclusion of this article will be made available by the authors, without undue reservation.

## Abbreviations

RT, remimazolam tosilate; ASA, american society of anesthesiologists; BMI, body mass index; GABAA, γ-aminobutyric acid A.
